# Protrudin-deficient mice manifest depression-like behavior with abnormalities in activity, attention, and cued fear-conditioning

**DOI:** 10.1186/s13041-020-00693-3

**Published:** 2020-11-10

**Authors:** Michiko Shirane, Hirotaka Shoji, Yutaka Hashimoto, Hiroyuki Katagiri, Shizuka Kobayashi, Toshiya Manabe, Tsuyoshi Miyakawa, Keiichi I. Nakayama

**Affiliations:** 1 Department of Molecular Biology, Graduate School of Pharmaceutical Sciences, Nagoya City University, Nagoya, Aichi Japan; 2 Division of Systems Medical Science, Institute for Comprehensive Medical Science, Fujita Health University, Toyoake, Aichi Japan; 3 Department of Molecular and Cellular Biology, Medical Institute of Bioregulation, Kyushu University, Fukuoka, Fukuoka Japan; 4 Division of Neuronal Network, Department of Basic Medical Sciences, Institute of Medical Science, University of Tokyo, Tokyo, Japan

**Keywords:** Protrudin, Knockout mouse, Behavior, Hyperactivity, Depression

## Abstract

Protrudin is a protein that resides in the membrane of the endoplasmic reticulum and is highly expressed in the nervous system. Although mutations in the human protrudin gene (*ZFYVE27*, also known as *SPG33*) give rise to hereditary spastic paraplegia (HSP), the physiological role of the encoded protein has been largely unclear. We therefore generated mice deficient in protrudin and subjected them to a battery of behavioral tests designed to examine their intermediate phenotypes. The protrudin-deficient mice were found to have a reduced body size and to manifest pleiotropic behavioral abnormalities, including hyperactivity, depression-like behavior, and deficits in attention and fear-conditioning memory. They exhibited no signs of HSP, however, consistent with the notion that HSP-associated mutations of protrudin may elicit neural degeneration, not as a result of a loss of function, but rather as a result of a gain of toxic function. Overall, our results suggest that protrudin might play an indispensable role in normal neuronal development and behavior.

## Introduction

Protrudin is an endoplasmic reticulum (ER)–resident protein that promotes neurite formation through regulation of endosome trafficking [1–8]. We have recently shown that protrudin and its binding partner PDZD8 cooperatively play an important role in the development of neuronal polarity and maintenance of neuronal integrity by mediating the tethering of endosomes to ER and consequent lipid transfer at the membrane contact sites that is essential for endosome maturation [9–11]. Loss of protrudin or PDZD8 thus impairs the establishment of neuronal polarity and gives rise to neurodegeneration in association with compromised endosome maturation.

Mutations of the human protrudin gene—*ZFYVE27*, also known as *SPG33* (spastic paraplegia gene 33)—give rise to hereditary spastic paraplegia (HSP), an axonopathy characterized by progressive spasticity and weakness of the lower limbs due to degeneration of the long axons of corticospinal tract motor neurons [12–16]. Most HSP-causative genes encode proteins that interact with each other and which harbor a hairpin domain that wedges in the ER membrane and increases its curvature, resulting in formation of the tubular ER network [17–21]. Given that most individuals with HSP are heterozygous for mutations of SPGs that are inherited in a dominant manner, mere loss of function of the encoded proteins may not account for disease pathogenesis. We also previously showed that expression of the G191V mutant of protrudin, which has been identified in a subset of HSP patients, confers an increased susceptibility of cells to ER stress [16]. These lines of indirect evidence suggest that a gain of toxic function such as abnormal protein aggregation in the ER may elicit ER stress and neurodegeneration, and thereby account for HSP pathogenesis. On the other hand, as mentioned above, loss of protrudin also results in defective endolysosome homeostasis and neurodegeneration at the cellular level [9, 22].

To explore the physiological function of protrudin, as well as its pathological role in HSP, we generated mice lacking protrudin and subjected them to a series of behavioral tests. The protrudin-deficient mice did not show any signs of HSP, but they manifested a variety of behavioral abnormalities related to activity, attention, and fear-conditioning memory as well as a depression-like phenotype.

## Results

### Generation of protrudin-deficient mice

**Fig. 1:**
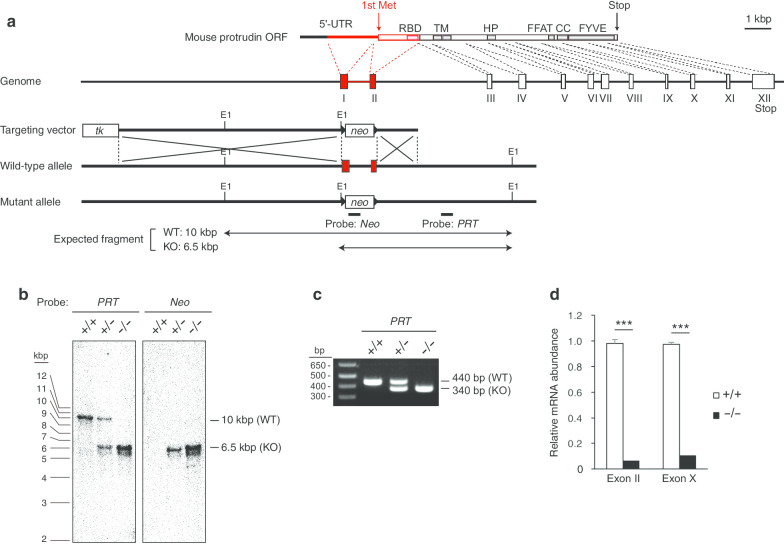
Gene targeting of the mouse protrudin locus. **a** Schematic representation of the domain structure for the protrudin open reading frame (ORF), the genomic structure of the protrudin gene locus, the targeting vector, the wild-type (WT) allele, and the mutant (KO) allele after homologous recombination. A genomic fragment including exons I and II of the protrudin coding region (red) was replaced with a phosphoglycerate kinase (PGK)–neo cassette. *UTR* untranslated region, *RBD* Rab11 binding domain, *TM* transmembrane domains, *HP* hairpin domain, *FFAT* two phenylalanines in an acidic tract, *CC* coiled-coil domain, *FYVE* Fab1-YOTB-Vac1-EEA1 domain, *tk* thymidine kinase gene, *neo* neomycin resistance gene, *E1* EcoR1 site. **b** Southern blot analysis of EcoRI-digested genomic DNA from mice of the indicated protrudin genotypes (*PRT*) with the probes shown in (**a**). The 10- and 6.5-kbp bands corresponding to the WT and KO alleles, respectively, are indicated. **c** PCR analysis of genomic DNA from mice of the indicated protrudin genotypes. The 440- and 340-bp bands corresponding to the WT and KO alleles, respectively, are indicated. **d** Quantitative RT-PCR analysis of mRNA derived from exons II or X of the protrudin gene in mouse embryos of the indicated protrudin genotypes. Data are means + SEM (*n* = 3). ***P < 0.001 (Student’s *t* test)

For generation of protrudin-deficient mice, we designed a targeting vector to delete the genomic region containing the first ATG codon of the protrudin gene (Fig. 1a). We introduced this vector into mouse embryonic stem cells (ESCs) by transfection and eventually obtained cells harboring the expected mutant allele. The mutant ESCs were injected into C57BL/6 J mouse blastocysts, and chimeric males that transmitted the mutant allele in the germ line were identified. Heterozygous mice were intercrossed to produce homozygous mutant animals, hereafter referred to as *PRT*^–/–^ mice. We verified the homologous recombination event by Southern blot analysis (Fig. 1b) and polymerase chain reaction (PCR) analysis (Fig. 1c) of the mutant mice. Furthermore, quantitative reverse transcription (RT)–PCR analysis of mRNA derived from the regions corresponding to exon II or exon X of the protrudin gene revealed that the abundance of such transcripts was greatly reduced or almost undetectable in *PRT*^–/–^ mice (Fig. 1d). Genotyping analysis revealed a normal Mendelian ratio of mutant offspring produced from the breeding of *PRT*^+*/–*^ mice (data not shown). *PRT*^–/–^ mice also appeared normal and did not manifest signs of spastic paraplegia or a gait disorder similar to those of individuals with HSP. Signs of defects in motor neurons associated with these conditions should have been apparent in the rotarod test, balance-beam test, and open-field test, but so such signs were detected (see below).

### Normal general health and neurological phenotype of protrudin-deficient mice

We performed a battery of behavioral tests to elucidate the possible influence of protrudin ablation on physiological endophenotypes with the use of 22 wild-type (WT) and 17 *PRT*^–/–^ mice, both of which had been backcrossed to the C57BL/6 J background for > 10 generations and were obtained as a single cohort by heterozygote intercrossing.

**Fig. 2:**
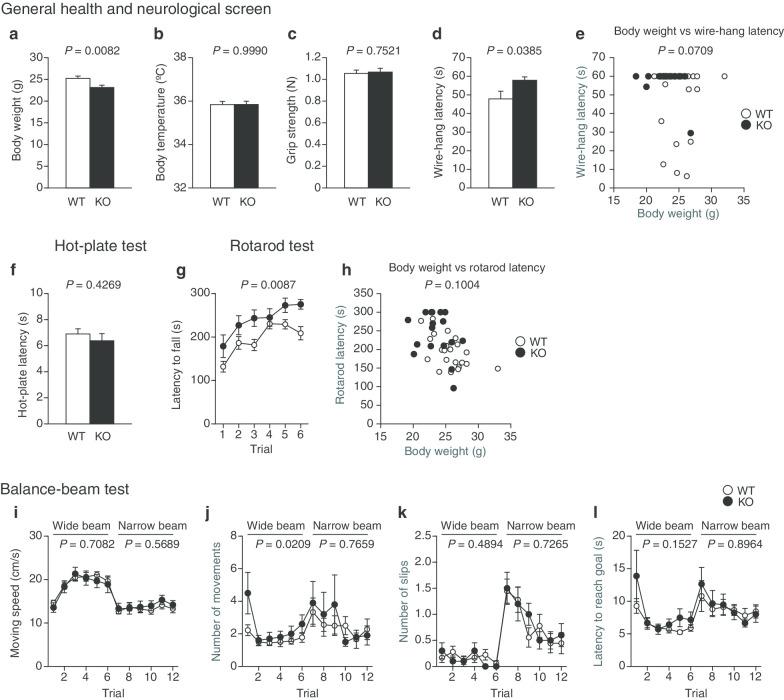
General health and neurological screen of protrudin-deficient mice. **a**, **b**, Body weight (**a**) and body temperature (**b**) of male WT and *PRT*^–/–^ (KO) mice at 12 to 17 weeks of age. **c** Grip strength. **d** Latency to fall in the wire-hang test. **e**, Scatter plot of body weight and wire-hang latency. **f** Latency of the first paw response in the hot-plate test of pain sensitivity. **g** Latency to fall from the rotating rod in the rotarod test for motor function. **h** Scatter plot of body weight and rotarod latency. **i**–**l** Movement speed (**i**), number of movements (**j**), number of slips (**k**), and latency to reach the goal (**l**) in the balance-beam test for coordinated movement and sense of balance. All data are means ± SEM (WT mice, *n* = 22 in **a**–**h** and *n* = 18 in **i**–**l**; *PRT*^–/–^ mice, *n* = 17 in **a**–**h** and *n* = 10 in **i**–**l**). *P* values for differences between genotypes were determined by Student's *t* test (**a**, **b**, **f**), Wilcoxon rank sum test (**c**, **d**), ANCOVA (**e**, **h**), or two-way repeated-measures ANOVA (**g**, **i**–**l**). The results of all statistical analysis are provided in Additional file 1: Table S1

We first examined the general health of these mice and performed a neurological screen. *PRT*^–/–^ mice showed a reduced body weight (*P* = 0.0082) (Fig. 2a) but had a normal body temperature (Fig. 2b). Grip strength did not differ significantly between WT and *PRT*^–/–^ mice (Fig. 2c), whereas the wire-hang test revealed that muscle strength was greater in the mutant animals (*P* = 0.0385) (Fig. 2d). Analysis of covariance (ANCOVA) with body weight as a covariate, however, indicated that there was no genotype effect on wire-hang latency (Fig. 2e). Analysis of pain sensitivity with the hot-plate test revealed no abnormality in *PRT*^–/–^ mice (Fig. 2f). Motor coordination and motor learning were assessed with the rotarod test, in which the time for mice to fall from the rotating cylinder is measured. *PRT*^–/–^ mice showed an increased latency to falling compared with WT mice (*P* = 0.0087) (Fig. 2g), suggestive of enhanced motor function in the mutant animals. However, as in previous studies [23, 24], rotarod latency was found to be negatively correlated with body weight (Pearson correlation coefficient = – 0.5177, *P* = 0.0007). Given the possible influence of body weight on rotarod performance, we reanalyzed the results of the rotarod test by ANCOVA with body weight as a covariate, which revealed no genotype effect on rotarod latency (Fig. 2h). In addition, given that the increased latency for *PRT*^–/–^ mice was apparent in the first of the six trials, this difference was unlikely to be related to learning (Fig. 2g). Repeated two-way analysis of variance (ANOVA) showed no interaction between genotype and trial as well as no significant difference in changes from trial 1 to trial 6 between genotypes, suggesting that motor learning is indistinguishable between the two genotypes (Fig. 2g). We also assessed motor coordination and balance with the balance-beam test, in which mice walk along a wide or narrow beam that is elevated above the floor and leads to the goal of a dark box. Although there was no overall difference in motor coordination and balance between WT and *PRT*^–/–^ mice in this test (Fig. 2i–l), the mutant mice did not head straight for the goal when traversing the wide beam but rather stopped and sometimes turned in the opposite direction, resulting in an increased number of movements (*P* = 0.0209) (Fig. 2j). This increase in the number of movements may reflect a decline in goal-oriented behavior, attention, and concentration, rather than abnormal locomotor activity, in the mutant mice. Together, these various observations revealed that *PRT*^–/–^ mice are smaller than are their WT littermates, that they manifest no abnormalities in body temperature, muscle strength, pain sensitivity, and motor coordination or balance, but that they may be abnormal with regard to goal-directed behavior, attention, and concentration.

### No change in anxiety-related behavior in protrudin-deficient mice

**Fig. 3:**
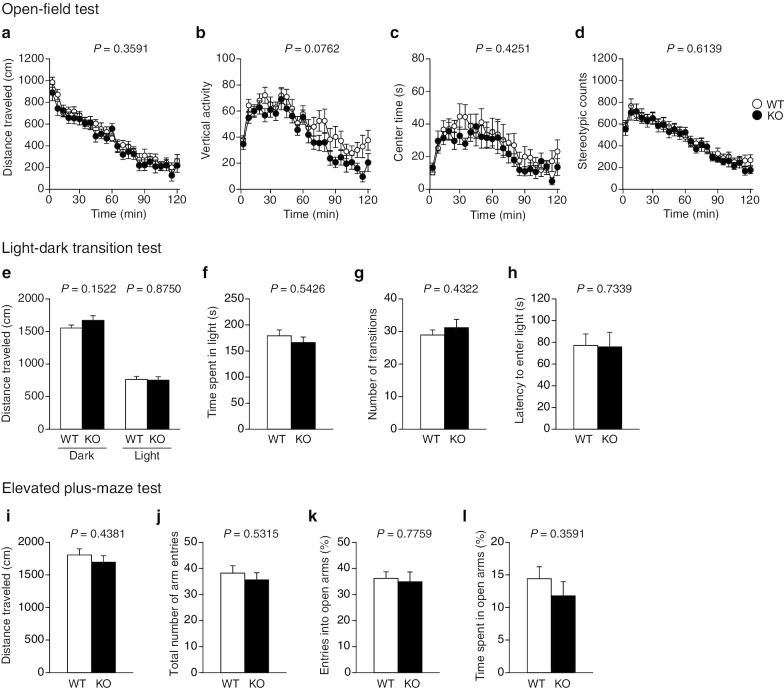
Anxiety-related behavior of protrudin-deficient mice. **a**–**d** Distance traveled (**a**), vertical activity (**b**), center time (**c**), and stereotypic counts (**d**) for the open-field test. **e**–**h** Distance traveled in the light and dark chambers (**e**), time spent in the light chamber (**f**), number of transitions between the light and dark chambers (**g**), and latency of entry into the light chamber (**h**) for the light–dark transition test. **i**–**l** Distance traveled (**i**), total number of arm entries (**j**), percentage of entries into the open arms (**k**), and percent time spent in the open arms (**l**) for the elevated plus-maze test. All data are means ± SEM (WT mice, *n* = 22; *PRT*^–/–^ mice, *n* = 17 for **a**–**h** and *n* = 15 for **i**–**l**). *P* values for differences between genotypes were determined by two-way repeated-measures ANOVA (**a**–**d**) Student's *t* test (**e**, **g**, **i**–**l**), or Wilcoxon rank sum test (**f**, **h**). The results of all statistical analysis are provided in Additional file 1: Table S1

To assess anxiety-related behavior, we performed the open-field test, light–dark transition test, and elevated plus-maze test. In the open-field test, distance traveled (Fig. 3a), vertical activity (Fig. 3b), time spent in the center space (Fig. 3c), and stereotypic counts (Fig. 3d) did not differ significantly between WT and *PRT*^–/–^ mice. The light–dark transition test also revealed no significant difference in distance traveled (Fig. 3e), time spent in the light chamber (Fig. 3f), number of transitions between the light and dark chambers (Fig. 3g), and latency to enter the light chamber (Fig. 3h) between the two genotypes. In the elevated plus-maze test, we did not detect any differences in distance traveled (Fig. 3i), total number of arm entries (Fig. 3j), percentage of entries into the open arms (Fig. 3k), and time spent in the open arms (Fig. 3l) between *PRT*^–/–^ mice and WT mice. On the basis of these collective results, we concluded that WT and *PRT*^–/–^ mice do not differ in anxiety-related behavior.

### Social interaction of protrudin-deficient mice

**Fig. 4:**
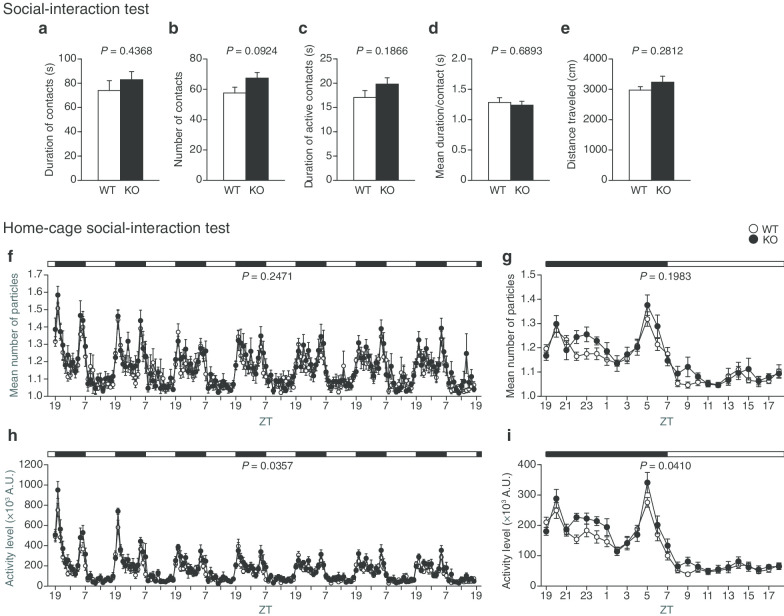
Social interaction of protrudin-deficient mice. **a**–**e** Duration of contacts (**a**), number of contacts (**b**), duration of active contacts (**c**), mean duration per contact (**d**), and distance traveled (**e**) in the social-interaction test. **f**–**i** Mean number of particles calculated for each hour over 7 days (**f**) and averaged over the last 3 days (**g**) as well as mean activity level (A.U., arbitrary units) for each hour over 7 days (**h**) and averaged over the last 3 days (**i**) in the home-cage social-interaction test. Time points are indicated in Zeitgeber time (ZT). All data are means ± SEM (WT mice, *n* = 11; *PRT*^–/–^ mice, *n* = 8). *P* values for differences between genotypes were determined with Student's *t* test (**a**–**e**) or by two-way repeated-measures ANOVA (**f**–**i**). The results of all statistical analysis are provided in Additional file 1: Table S1

In the social-interaction test that assesses social behavior in a novel environment, two mice are put in a cage and their behavior is monitored for a short time (10 min). The total duration of contacts (Fig. 4a), total number of contacts (Fig. 4b), duration of active contacts (Fig. 4c), mean duration per contact (Fig. 4d), and distance traveled (Fig. 4e) did not differ significantly between WT and *PRT*^–/–^ mice. In the home-cage social-interaction test, two mice are placed in a cage and monitored for 7 days, with their activity levels and mean number of contacts being measured. Mice of the two genotypes did not show any significant difference in the mean number of particles (two particles indicated that the mice were not in contact with each other; one particle indicated contact between the two mice) per hour either over 7 days (Fig. 4f) or averaged over the last 3 days (Fig. 4g). However, *PRT*^–/–^ mice showed an increase in mean activity both over 7 days (*P* = 0.0357) (Fig. 4h) and averaged over the last 3 days (*P* = 0.0410) (Fig. 4i) compared with WT mice, suggesting that the mutant animals are more active than WT mice in a familiar environment.

### Normal sociability and social-novelty preference in protrudin-deficient mice

**Fig. 5:**
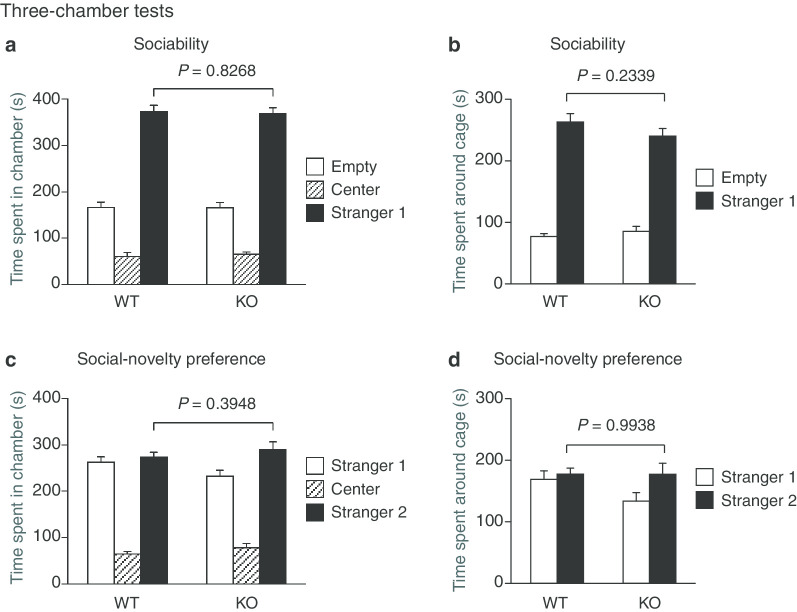
Sociability and social-novelty preference of protrudin-deficient mice. **a**, **b** Time spent in the chamber with an empty cage, the center chamber, and the chamber with a cage containing a stranger mouse (stranger 1) (**a**) as well as time spent around each cage (**b**) in the three-chamber sociability test. **c**, **d** Time spent in the chamber with the cage containing stranger 1, the center chamber, and the chamber with the cage now containing a novel unfamiliar mouse (stranger 2) (**c**) as well as time spent around each cage (**d**) in the three-chamber test of social-novelty preference. All data are means + SEM (WT mice, *n* = 22; *PRT*^–/–^ mice, *n* = 17). *P* values for differences between genotypes were determined with Student's *t* test (**a**–**c**) or Welch *t* test (**d**). The results of all statistical analysis are provided in Additional file 1: Table S1

To assess sociability, we placed a test mouse in a three-chambered box containing an empty cage or a cage including an unfamiliar mouse (stranger 1) in the end chambers and then monitored the location of the test animal. Neither the time spent in each chamber nor that spent around each cage differed significantly between WT and *PRT*^–/–^ mice (Fig. 5a, b). In the social-novelty preference test, another unfamiliar mouse (stranger 2) was then placed in the empty cage and the preference of the test mouse for the new stranger mouse was examined. Again, the time spent in each chamber and the time spent around each cage did not differ significantly between WT and *PRT*^*–/–*^ mice (Fig. 5c, d). Collectively, these results suggested that *PRT*^–/–^ mice are normal with regard to social behavior.

### Depression-like behavior and a reduced startle response in protrudin-deficient mice

**Fig. 6:**
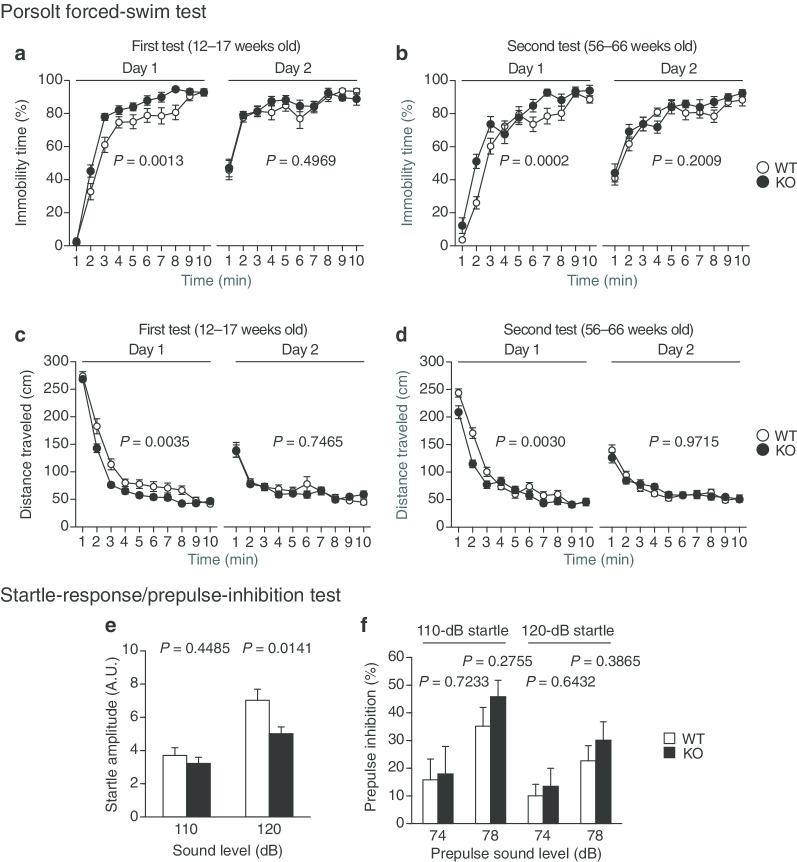
Depression-like behavior, startle response, and prepulse inhibition in protrudin-deficient mice. **a**–**d** Percentage immobility time (**a**, **b**) and distance traveled (**c**, **d**) on day 1 and day 2 for mice at 12 to 17 weeks of age (**a**, **c**) and the same mice at 56 to 66 weeks of age (**b**, **d**) in the Porsolt forced-swim test. **e**, **f** Startle amplitude (A.U., arbitrary units) (**e**) and prepulse inhibition (**f**) in the startle-response and prepulse-inhibition test. All data are means ± SEM (WT mice, *n* = 22; *PRT*^–/–^ mice, *n* = 17). *P* values for differences between genotypes were determined by two-way repeated-measures ANOVA (**a**–**d**) or Student's *t* test (**e**, for 110 dB; f, for 74–120 dB and 78–120 dB), Welch *t* test (**e**, for 120 dB), or Wilcoxon rank sum test (**f**, for 74–110 dB and 78–110 dB). The results of all statistical analysis are provided in Additional file 1: Table S1

WT and *PRT*^–/–^ mice at 12 to 17 and 56 to 66 weeks of age were subjected to the Porsolt forced-swim test on two consecutive days. Immobility time on day 1 was markedly longer for *PRT*^–/–^ mice than for WT mice at 12 to 17 weeks of age (*P* = 0.0013) (Fig. 6a), whereas no such difference was apparent on day 2. Similar results were obtained for mice at 56 to 66 weeks of age (*P* = 0.0002 for day 1) (Fig. 6b). Distance traveled on day 1 was also shorter for *PRT*^*–/–*^ mice than for WT mice at both 12 to 17 (*P* = 0.0035) (Fig. 6c) and 56 to 66 (*P* = 0.0030) (Fig. 6d) weeks of age. These results were suggestive of depression-like behavior in *PRT*^*–/–*^ mice.

We performed the acoustic startle-response and prepulse-inhibition test to assess the attention of the mutant mice. The startle response to a single loud sound was significantly reduced in *PRT*^–/–^ mice compared with WT mice (*P* = 0.0141 for the 120-dB stimulus) (Fig. 6e). In contrast, no significant difference in prepulse inhibition between the two genotypes was observed (Fig. 6f), suggesting that *PRT*^–/–^ mice have a deficit in the startle response to a loud sound and in attention ability. The startle response to a single sound at 110 dB did not differ between WT and *PRT*^–/–^ mice (Fig. 6e). Furthermore, for both genotypes, the extent of prepulse inhibition for the pairing of 78 and 110 dB was higher than that for the pairing of 74 and 110 dB. Although the difference in prepulse stimulation between trials was only 4 dB, *PRT*^–/–^ mice were able to segregate and respond to this sound difference in the same way as WT mice. These results suggested that *PRT*^–/–^ mice are unlikely to have a hearing impairment.

### Impaired fear-conditioning memory in protrudin-deficient mice

**Fig. 7:**
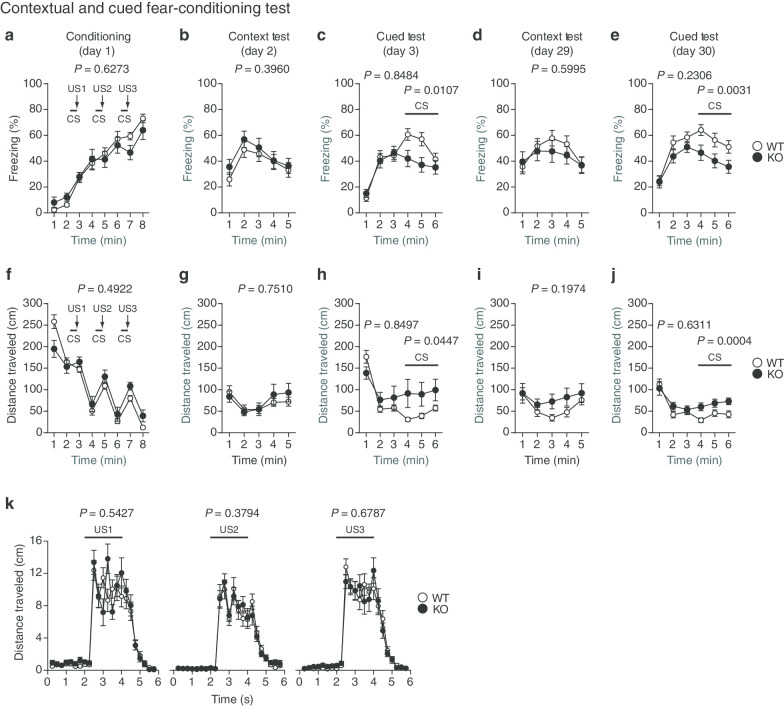
Contextual and cued fear memory of protrudin-deficient mice. **a**–**j** Percent freezing time (**a**–**e**) and distance traveled (**f**–**j**) during conditioning (**a**, **f**), in a context test performed 1 day (**b**, **g**) or 28 days (**d**, **i**) after conditioning, and in a cued test with altered context performed 2 days (**c**, **h**) or 29 days (**e**, **j**) after conditioning in the contextual and cued fear-conditioning test. Mice were presented three times with white noise as a conditioned stimulus (CS) for 30 s (horizontal black bars) followed by foot shock as an unconditioned stimulus (US) for the last 2 s of the conditioned stimulus (vertical arrows) during the conditioning session. **k** Shock sensitivity assessed on the basis of the distance traveled during and after exposure to the unconditioned stimulus. All data are means ± SEM (WT mice, *n* = 22; *PRT*^–/–^ mice, *n* = 17). *P* values for differences between genotypes were determined by two-way repeated-measures ANOVA (**a**–**k**). For the cued test, separate *P* values are shown for the first and second 3-min periods. The results of all statistical analysis are provided in Additional file 1: Table S1

For assessment of fear-related learning and memory, each mouse was placed in a chamber and presented three times with a conditioned stimulus of white noise followed by an unconditioned stimulus of mild foot shock (Fig. 7a, f). A contextual test followed by a cued test were then performed. Freezing time (Fig. 7b–e) and distance traveled (Fig. 7g–j) were monitored to evaluate contextual memory (Fig. 7b, d, g, i) and cued memory (Fig. 7c, e, h, j) at both a short interval (1 or 2 days) (Fig. 7b, c, g, h) and a long interval (28 or 29 days) (Fig. 7d, e, i, j) after conditioning. We confirmed that reactivity to the unconditioned stimulus was normal in *PRT*^–/–^ mice (Fig. 7k). Whereas there was no significant difference between the two genotypes in freezing time or distance traveled in the context test (Fig. 7b, d, g, i), *PRT*^–/–^ mice manifested a significantly reduced freezing time (Fig. 7c, e) and increased distance traveled (Fig. 7h, j) compared with WT mice in the cued test. These results suggested that *PRT*^–/–^ mice are normal with regard to contextual memory but are defective in cued fear memory.

### Normal spatial, reference, and working memory in protrudin-deficient mice

**Fig. 8:**
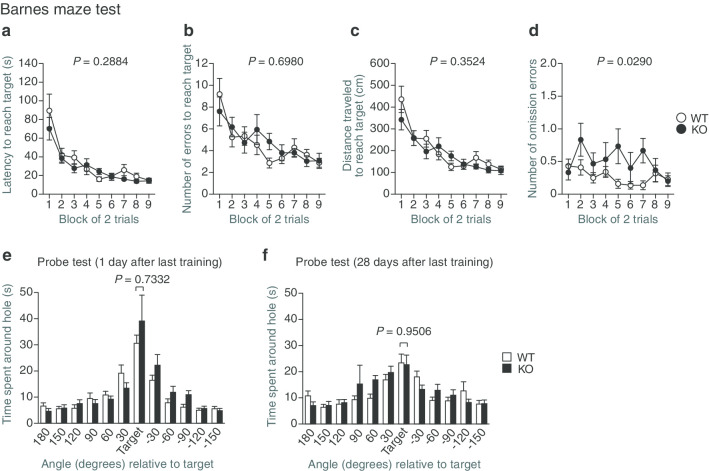
Spatial reference memory of protrudin-deficient mice assessed with the Barnes maze test. **a**–**d** Latency to reach the target hole (**a**) as well as the number of errors (**b**), distance traveled (**c**), and number of omission errors (**d**) before reaching the target hole during training sessions. **e**, **f** Time spent around each hole in probe trials conducted 1 day (**e**) and 28 days (**f**) after the last training session. All data are means ± SEM (WT mice, *n* = 22; *PRT*^–/–^ mice, *n* = 15). *P* values for differences between genotypes were determined by two-way repeated-measures ANOVA (**a**–**d**) or Wilcoxon rank sum test (**e**, **f**). The results of all statistical analysis are provided in Additional file 1: Table S1

**Fig. 9:**
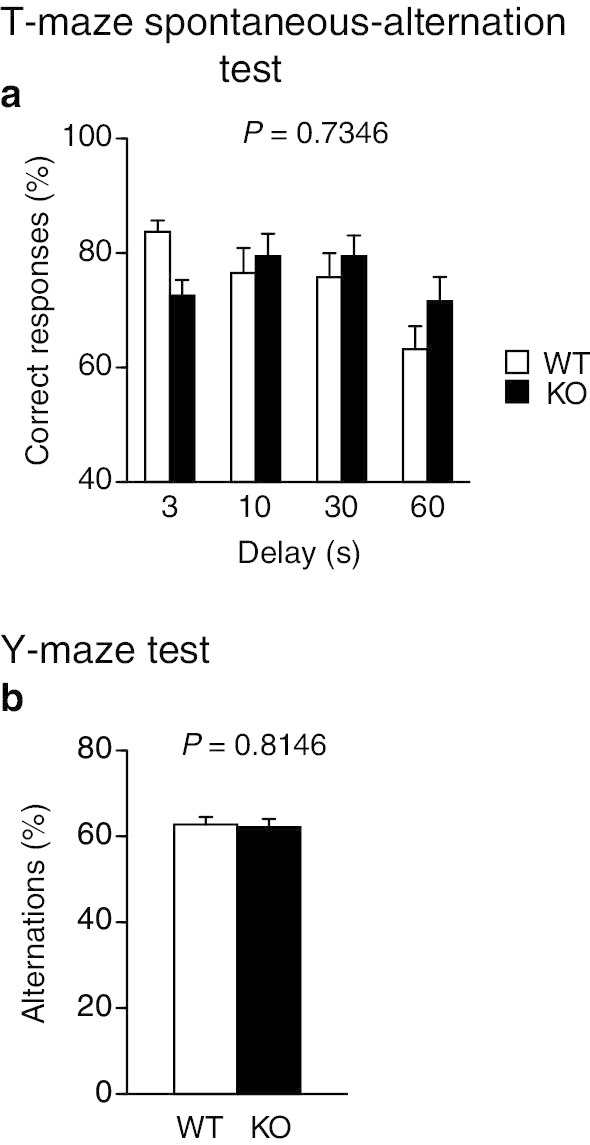
Working memory of protrudin-deficient mice. **a** Percent correct responses in the T-maze test. **b**, Percentage of alternations in the Y-maze test. All data are means + SEM (WT mice, *n* = 22; *PRT*^–/–^ mice, *n* = 17). *P* values for differences between genotypes were determined by two-way repeated-measures ANOVA (**a**) or Student's *t* test (**b**). The results of all statistical analysis are provided in Additional file 1: Table S1

To assess spatial learning and memory, we performed the Barnes maze test. Mice were trained to find a target box located under one of the holes in a maze for nine consecutive days (two trials per day, 18 trials in total). Neither latency to reach the target hole (Fig. 8a) nor the number of errors (Fig. 8b), or distance traveled (Fig. 8c), except for the number of omission errors (Fig. 8d), before reaching the target hole differed between WT and *PRT*^–/–^ mice. To evaluate spatial reference memory, we conducted probe trials without the escape box at 1 and 28 days after the last training trial. *PRT*^–/–^ mice showed normal memory performance in both probe trials (Fig. 8e, f). Finally, to assess working memory, we performed the T-maze spontaneous-alternation test and Y-maze test. The percentage of correct responses (Fig. 9a) or alternations (Fig. 9b), respectively, did not differ significantly between WT and *PRT*^–/–^ mice in these tests. Together, these results suggested that spatial learning, reference, and working memory are normal in *PRT*^–/–^ mice.

### Synaptic plasticity in the hippocampus of protrudin-deficient mice

**Fig. 10:**
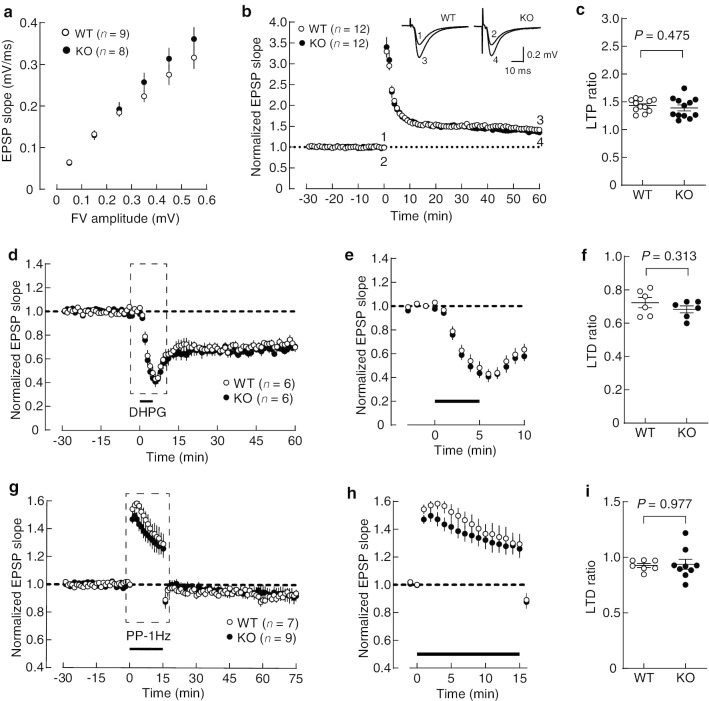
Electrophysiological analysis of the hippocampus of protrudin-deficient mice. **a** Input–output relation for AMPA receptor–mediated synaptic responses in acute hippocampal slices of WT and *PRT*^–/–^ mice. FV, fiber volley. **b** Time course of LTP induced by tetanic stimulation. A train of high-frequency stimulation was applied at time 0. Representative traces (averaged 10 consecutive responses) in the inset are EPSPs obtained at the times indicated by the numbers in the main panel. **c** Ratio of LTP at 60 min after tetanic stimulation. The *P* value was determined with Student’s unpaired *t* test. **d** Time course of LTD induced by DHPG application for 5 min (black bar). **e** Expanded view of the portions of the curves outlined by the dashed lines in **d**. **f** Ratio of LTD at 60 min after the start of DHPG application. The *P* value was determined with Student’s unpaired *t* test. **g** Time course of synaptic depression induced by low-frequency stimulation. PP-1 Hz, paired-pulse stimulation (interpulse intervals, 50 ms) at 1 Hz for 15 min (black bar). **h** Expanded view of the portions of the curves outlined by the dashed lines in **g**. **i** Ratio of depression at 60 min after the end of the 1-Hz stimulation. The *P* value was determined with Welch’s *t* test. All data are means ± SEM, with 6–12 values indicating the number of slices examined (each slice was from a different mouse)

We assessed functional synaptic plasticity at Schaffer collateral-commissural fiber synapses in the CA1 region of the hippocampus by electrophysiology with acute hippocampal slices. To evaluate basal synaptic transmission, we examined the input–output relation with the use of the extracellular field-potential recording technique. The slope of α-amino-3-hydroxy-5-methyl-4-isoxazolepropionic acid (AMPA) receptor–mediated excitatory postsynaptic potentials (EPSPs, output) in response to stimulation that evoked the same fiber volley amplitude (input) did not differ significantly between WT and *PRT*^–/–^ mice, indicating that the efficacy of basal synaptic transmission was normal in the mutant mice (Fig. 10a). Furthermore, long-term potentiation (LTP) induced by tetanic stimulation was intact in *PRT*^–/–^ mice (Fig. 10b). The ratio of LTP at 60 min after tetanic stimulation was indistinguishable between WT and *PRT*^–/–^ mice (Fig. 10c). We also examined synaptic depression. Long-term depression (LTD) induced by (s)-3,5-dihydroxyphenylglycine (DHPG) was also normal in *PRT*^–/–^ mice (Fig. 10d, e). The ratio of LTD at 60 min did not differ significantly between WT and *PRT*^–/–^ mice (Fig. 10f). Paired-pulse stimulation–induced LTD was also normal in *PRT*^–/–^ mice (Fig. 10g, h), with the LTD ratio at 60 min again not differing significantly between the WT and mutant animals (Fig. 10i). Together, these results suggested that synaptic plasticity in the hippocampus is intact in *PRT*^–/–^ mice.

## Discussion

We generated mice deficient in protrudin and subjected them to a battery of behavioral tests. Our results indicate that the *PRT*^–/–^ mice have a reduced body size, show increased activity, being hyperactive in a familiar environment, as well as manifest reduced goal-directed behavior, an attenuated startle response to a sound stimulus, an attention deficit, impaired fear-conditioning memory, and increased depression-like behavior compared with WT mice.

At present, it is difficult to directly link the known cellular functions of protrudin—such as regulation of ER morphology, endosome trafficking and maturation, and neuronal polarity—to the behavioral phenotype of *PRT*^–/–^ mice. One possible hypothesis is that protrudin regulates neurotransmitter release through its effects on ER and endosome homeostasis, with the result that certain neuronal circuits are defective in *PRT*^–/–^ mice. In particular, the increased activity, reduced attention, defect in cued fear memory, and depression-like behavior of *PRT*^–/–^ mice might be related to psychiatric conditions such as bipolar disorder and attention deficit–hyperactivity disorder in humans.

Although mutations of the protrudin gene give rise to HSP in humans, the detailed pathogenesis of this condition remains largely unknown. In the present study, we found no signs of spastic paraplegia in *PRT*^–/–^ mice, suggesting that mutations of the protrudin gene in individuals with HSP likely confer a gain of toxic function rather than a loss of function on the encoded protein. Such mutations might thus promote intracellular aggregation of protrudin in neurons and thereby elicit neurodegeneration. This notion is consistent both with the fact that most individuals with HSP are heterozygous for mutations of SPGs, with the mutations being inherited in a dominant manner, as well as with our previous finding that expression of the protrudin(G191V) mutant increases the susceptibility of cells to ER stress [16]. Generation and characterization of mice expressing protrudin(G191V) should shed light on this issue.

We have recently shown that protrudin forms a complex with PDZD8 and promotes endosome maturation by facilitating lipid transfer from the ER to endosomes [9]. The amount of PDZD8 was markedly reduced in the brain of *PRT*^–/–^ mice and, in a reciprocal manner, the abundance of protrudin was greatly diminished in the brain of mice deficient in PDZD8. These results suggest that protrudin and PDZD8 mutually stabilize each other. It is thus possible that protrudin and PDZD8 are cotranslated and form a stable complex at the ER membrane in neurons in the brain, whereas the individual proteins undergo rapid degradation by the proteasome when expressed alone. Given the reciprocal dependence of the stability of these molecules on each other, the phenotypes apparent in *PRT*^–/–^ mice might be attributable, at least in part, to the reduced expression of PDZD8. Depletion of protrudin or PDZD8 in primary neurons of the mouse brain results in abnormal endosome maturation, impaired neuronal polarity, and increased neuronal degeneration. Such phenotypes might account for the abnormal behavioral characteristics of *PRT*^–/–^ mice, including the motor hyperactivity, attention and fear memory deficits, and depressive behavior. The behavioral phenotypes of PDZD8-deficient mice when determined might thus be expected to be similar to those of *PRT*^–/–^ mice. It is of note that PDZD8 mutation is a risk factor for posttraumatic stress disorder in humans [25], and further investigation into the function of the protrudin-PDZD8 complex might therefore shed light on the etiology of this disorder.

## Methods

### Generation of protrudin-deficient mice

The targeting vector for protrudin was constructed by replacement of a 1.2-kbp fragment of genomic DNA containing exons I and II of the protrudin gene with a PGK-neo-poly(A)-loxP cassette. Maintenance, transfection, and selection of mouse ESCs were performed as described previously [26]. Mutant ESCs were microinjected into C57BL/6 J blastocysts, and resulting male chimeras were mated with C57BL/6 J mice. Heterozygous offspring were intercrossed to produce homozygous mutant animals and littermate controls. All mice in this study were backcrossed to the C57BL/6 J background for > 10 generations.

### Genotyping

The homologous recombination event and germ-line transmission of the mutant allele were confirmed by Southern blot analysis, in which DNA from mouse tail was digested with EcoRI, transferred to a nylon membrane, and then exposed to probes shown in Fig. 1a. Animals were genotyped by PCR with the primers 5′-TTGTCTCTGCGTTCTCACGGCAAG-3′, 5′-TCCTGCCACCCAAGAGATGACTCG-3′, and 5′-TCCAGACTGCCTTGGGAAAAGCGC-3'.

### Quantitative RT-PCR analysis

Total RNA isolated from mouse brain with the use of an RNeasy Lipid Tissue Mini Kit (Qiagen) was subjected to RT with a QuantiTect Reverse Transcription Kit (Qiagen) [27, 28]. The resulting cDNA was subjected to real-time PCR analysis with the use of SYBR Premix Ex Taq (Takara) and specific primers in a StepOnePlus Real-Time PCR System (Applied Biosystems). The primer sequences (sense and antisense, respectively) were 5′-GTCCACCTGCCCCTACCAAG-3′ and 5′-TTGTAGGACAGGACCAGGTTG-3′ for exon II and 5′-CGGCTTCGCAAGCGTTA-3′ and 5′-GGCACAGCCTGCACAATTC-3′ for exon X. The amount of each target mRNA was calculated and normalized by that of GAPDH mRNA. The primer sequences for GAPDH (sense and antisense) were 5′-CATGGCCTTCCGTGTTCCTA-3′ and 5′-GCGGCACGTCAGATCCA-3'.

### General protocol for behavioral tests

WT and *PRT*^–/–^ mice were group-housed (three or four animals per cage) in a room with a 12-h-light, 12-h-dark cycle (lights on at 7 a.m. and off at 7 p.m.) and with access to food and water ad libitum. Behavioral tests were performed between 9 a.m. and 6 p.m. with male mice at 12 to 17 weeks of age (or 56–66 weeks for the second trial of the Porsolt forced-swim test) as described previously [29–33], unless indicated otherwise. Each apparatus was cleaned with hypochlorite solution before testing of each animal in order to prevent bias due to olfactory cues.

### Neurological screen

Mice were subjected to physical assessment, including measurement of rectal temperature and body weight. A wire-hang test apparatus (O’Hara & Co., Tokyo, Japan) was used to assess muscular strength. The apparatus consists of a box (21.5 by 22 by 23 cm) with a wire-mesh grid (10 by 10 cm) on top that can be inverted. Mice were placed on the wire mesh, which was then inverted, causing the animal to grip the wire. The latency to falling was recorded, with a 60-s cutoff time. For assessment of forelimb grip strength, a mouse was held by its tail and lifted so that its forepaws could grasp the wire grid of a grip-strength meter (O’Hara & Co.). It was then gently pulled backward by the tail until it released the grid. The peak force applied by the forelimbs was recorded. Each mouse was tested three times, and the highest value was used for statistical analysis.

### Hot-plate test

Sensitivity to a painful stimulus was assessed with the hot-plate test. Mice were placed on a hot plate maintained at 55.0° ± 0.2 °C and with a black anodized aluminum surface (Columbus Instruments, Columbus, OH). The latency to the first paw response (foot shake or paw lick) was recorded, with a cutoff time of 15 s.

### Rotarod test

Motor coordination and balance were evaluated with the rotarod test. Each mouse was placed on a rotating rod (Accelerating Rotarod; UGO Basile, Varese, Italy), and the latency to falling off the rod (the time that each animal was able to maintain its balance on the rod) during its acceleration from 4 to 40 rpm over 5 min was measured in three trials per day over two consecutive days. The mice were subjected to this test without any pretest training.

### Balance-beam test

The balance-beam test apparatus consisted of a straight beam (100 cm in length) placed horizontally 50 cm above the floor and with a dark box placed at one end (O’Hara & Co.). Each mouse was placed on the other end of the beam from the box and allowed to traverse it in order to reach the box, with a 90-s cutoff time. Twelve trials were conducted in total, six trials with a wide beam (2.8 cm in diameter) followed by six trials with a narrow beam (1.1 cm in diameter). The moving speed, number of movement initiations, number of paw slips, and latency to reach the box were determined with the use of ImageBT software (see “Data analysis” section below).

### Open-field test

Each mouse was placed in the corner of an open-field apparatus that consisted of a transparent Plexiglas chamber (40 by 40 by 30 cm) with a white plastic floor (Accuscan Instruments, Columbus, OH) and which was illuminated at 100 lx. The total distance traveled, vertical activity (rearing, measured by counting the number of photobeam interruptions), time spent in the central area (20 by 20 cm), and beam-break counts for stereotyped behavior were recorded over 120 min.

### Light–dark transition test

The apparatus for the light–dark transition test consisted of a cage with a white floor made of PVC (21 by 42 by 25 cm) that was divided into two sections of equal size by a partition with a door (O’Hara & Co.). The walls and roof of one chamber were made of white plastic and the chamber was brightly illuminated (390 lx), whereas the walls and roof of the other chamber were made of black plastic and it was maintained dark (2 lx). Mice were placed in the dark chamber and allowed to move freely between the two rooms with the door open for 10 min. The distance travelled in both chambers, the number of transitions between the two chambers, the time spent in each chamber, and the latency to enter the light chamber were recorded with the use of ImageLD software.

### Elevated plus-maze test

The apparatus (O’Hara & Co.) consisted of two open arms (25 by 5 cm) and two enclosed arms of the same size with 15-cm-high transparent walls, and the arms were connected by a central square (5 by 5 cm). The arms and central square were made of white plastic plates and were elevated to a height of 55 cm above the floor. The likelihood of animals falling from the apparatus was minimized by the attachment of 3-mm-high plastic ledges to the open arms. Arms of the same type were arranged on opposite sides of the apparatus. Each mouse was placed in the central square of the maze facing one of the closed arms, and its behavior was recorded over 10 min. The total distance travelled, total number of arm entries, percentage of entries into the open arms, and percentage of time spent in the open arms were measured with the use of ImageEP software. Data for two mutant mice that fell from the maze were excluded from analysis.

### Social-interaction test in a novel environment

Two mice of the same genotype that had been housed in different cages were placed together in a box (40 by 40 by 30 cm) (O’Hara & Co.) and allowed to explore freely for 10 min. Analysis was performed automatically with the use of ImageSI software. Images were captured at a rate of three frames per second, and the distance traveled between two successive frames was determined for each mouse. If the two mice contacted each other and the distance traveled by them was > 10 cm, then the behavior was considered an active contact. The total duration of contacts, total number of contacts, total duration of active contacts, mean duration per contact, and total distance traveled were measured.

### Social-interaction test in the home cage

The home cage–monitoring system consisted of a home cage (25 by 15 by 23.5 cm, interior dimensions) and a cage top equipped with an infrared video camera (O’Hara & Co.). Two mice of the same genotype that had been housed separately were placed together in the home cage. Images from the cage were captured at a rate of one frame per second. Social interaction was measured by counting the number of particles detected in each frame (two particles indicated that the mice were not in contact with each other, and one particle indicated contact between the two mice). The activity level of the mice was also measured by quantifying the number of pixels that changed between each pair of successive frames. The mean number of particles and mean activity level were calculated over 1-h intervals for 7 days, with the values for the last 3 days being averaged for analysis of social behavior in a familiar environment. Analysis was performed automatically with the use of ImageHC software.

### Three-chamber tests of sociability and social-novelty preference

The testing apparatus consisted of a rectangular, three-chambered box with a lid fitted with a video camera (O’Hara & Co.). The dividing walls of the box were made of transparent plastic, with small square openings (5 by 3 cm) allowing access to each chamber (20 by 40 by 47 cm). A small round wire cage (9 cm in diameter and 11 cm in height, with vertical bars 0.5 cm apart) was located in a corner of the left and right chambers. The test mice were first placed in the middle chamber and allowed to explore the entire apparatus for 10 min. It was then transferred to a clean holding cage, while a male C57BL/6 J mouse (stranger 1) that had had no prior contact with the test mouse was enclosed in one of the wire cages. The location of stranger 1 in the left versus right side chamber was systematically alternated between trials. The test mouse was then returned to the middle chamber of the test box and allowed to explore for 10 min (sociability test). After the test session, the test mouse was again placed in the holding cage, and a second unfamiliar male C57BL/6 J mouse (stranger 2) was enclosed in the remaining empty wire cage. The test mouse was returned to the middle chamber of the test box and now had a choice to explore either the already-investigated unfamiliar mouse or the novel unfamiliar mouse for 10 min (social-novelty preference test). The time spent in each chamber and the time spent around each cage were automatically measured from images with the use of ImageCSI software.

### Porsolt forced-swim test

A Plexiglas cylinder (20 by 10 cm) (O’Hara & Co.) filled with hypochlorite solution at 21° to 23 °C up to a height of 7.5 cm was placed in a white plastic chamber (44 by 32 by 49 cm, inside dimensions) (O’Hara & Co.). Each mouse was placed in the cylinder, and its immobility time was recorded over a 10-min test period. Images were captured at a rate of two frames per second with a video camera. For each pair of successive frames, the area (number of pixels) within which the mouse moved was measured. When this area was below a certain threshold, the mouse was judged to be immobile. When the area equaled or exceeded the threshold, the mouse was considered to be moving. The optimal threshold was determined by adjustment based on the degree of immobility measured by human observation. Immobility lasting < 2 s was not included in the analysis. Data acquisition and analysis were performed automatically with the use of ImageTS/PS software.

### Startle-response and prepulse-inhibition test

A startle-reflex measurement system (O’Hara & Co.) was used to measure an acoustic startle response (ASR) elicited by a loud stimulus as well as prepulse inhibition of the startle response (PPI). Mice were placed in a PVC plastic cylinder. They were left undisturbed for 10 min and then subjected to test trials consisting of six trial types: two types of startle stimulus–only trial and four types of PPI trial. White noise of 110 or 120 dB (duration of 40 ms) was used as the startle stimulus for all trial types. A prepulse stimulus with an intensity of 74 or 78 dB (duration of 20 ms) was presented 100 ms before the onset of the startle stimulus. The four combinations of prepulse and startle stimuli were thus 74 and 110, 78 and 110, 74 and 120, and 78 and 120 dB. Mice were subjected to six blocks of the six trial types in a pseudorandom order such that each trial type was presented once within each block. The average intertrial interval was 15 s (range, 10–20 s). The startle response was recorded for 400 ms beginning with the onset of the startle stimulus. The peak startle amplitude was used as a dependent variable. The background noise level was 70 dB during all the test sessions. Percent PPI was calculated for each mouse according to the following formula: PPI (%) = 100 × {1 – [(ASR amplitude in prepulse + startle trial)/(ASR amplitude in startle stimulus–only trial)]}.

### Contextual and cued fear-conditioning test

For assessment of fear-related learning and memory, a mouse was placed in and allowed to explore freely for 2 min a plastic chamber consisting of white lateral and transparent front, rear, and top surfaces (33 by 25 by 28 cm) and with a stainless-steel grid floor (bars 0.2 cm in diameter, spaced 0.5 cm apart) (O’Hara & Co.). A conditioned stimulus (CS) of 55-dB white noise was presented for 30 s and was followed by a mild foot shock (0.3 mA), which was administered during the last 2 s of the CS and served as the unconditioned stimulus (US). Two more CS-US pairings were presented with an interval of 2 min between each pair. A context test was conducted in the same chamber for 5 min both 1 and 28 days after conditioning. Two and 29 days after conditioning, a cued test with an altered context was performed for 6 min in a triangular chamber (33 by 29 by 32 cm) that was made of white plastic walls and floor and was located in a different sound-attenuating room. In the cued test, the mouse was allowed to move freely for 3 min and was then exposed to the auditory stimulus (55-dB white noise) for 3 min. In each test, the percent freezing time and distance traveled were calculated automatically with the use of ImageFZ software. After each conditioning and context test, the plastic surfaces and grid floor of the chamber were wiped with hypochlorite solution and 65% ethanol, respectively. After each cued test, the walls and floor of the chamber were cleaned with hypochlorite solution. This experiment—including data acquisition, control of stimuli (white noise and foot shock), and data analysis performed automatically with ImageFZ software—was conducted as described previously [34]. In brief, the test chamber is equipped with a ceiling-mounted video camera connected to a computer for monitoring of mouse behavior. Images were captured at a rate of one frame per second. For each successive frame, the amount of area (pixels) within which the mouse moved was measured. When this area was below a certain threshold, the behavior was judged as “freezing.” When the area equaled or exceeded the threshold, the behavior was judged as “nonfreezing.” The optimal threshold (number of pixels) for judgment of freezing was determined by adjustment based on the degree of freezing measured by human observation.

### Barnes maze test

The Barnes maze test was performed on “dry land,” a white circular surface with a diameter of 100 cm and with 12 holes equally spaced around the perimeter (O’Hara & Co.). The circular open field was elevated 75 cm from the floor. The apparatus was illuminated by fluorescent lights mounted on the ceiling of a sound-attenuating room, with an illumination level of ~ 850 lx in the center of the field. A variety of fixed extramaze clues surrounded the apparatus. A black Plexiglas escape box (17 by 13 by 7 cm) was located under one of the holes, designated the target hole, which is analogous to the hidden platform in the Morris water-maze task. The location of the target was consistent for a given mouse but was randomized across mice. In a training session, the mouse was placed in the center of the field and allowed to explore the maze freely. After it had entered the target hole, the mouse was left undisturbed in the escape box for 30 s. The training session was conducted in two trials each day for nine consecutive days. The maze was rotated daily, with the spatial location of the target remaining unchanged with respect to the visual room cues in order to prevent bias based on olfactory or proximal cues within the maze. The latency to reach the target hole, number of errors before reaching the target hole, distance traveled to reach the target hole, and number of omission errors (defined by a visit to the target hole without subsequent entry into the escape box) were automatically recorded with the use of ImageBM software. One and 28 days after the last training session, probe trials were conducted without the escape box for 180 s in order to assess spatial reference memory. In the probe trials, the time spent around each hole was measured.

### T-maze test

The spontaneous-alternation task was conducted with an automatic T-maze apparatus (O’Hara & Co.) constructed of white plastic runways with 25-cm-high walls. The maze is partitioned into six areas by sliding doors that can be opened downward: stem of the T, straight runway, left and right arms, and connecting passageways from the arms to the stem of the T. Each mouse was subjected to sessions consisting of 10 trials per day (cutoff time, 50 min) for 3 days. Each trial consisted of a forced choice followed by a free choice. In the forced-choice run, the mouse was forced to enter either the left or right arm of the T-maze and was kept in the arm for 10 s. A free-choice run in which the mouse was allowed to choose one of the arms was then performed after a delay of 3, 10, 30, or 60 s. Choice by the mouse of the arm opposite that selected during the forced-choice run was considered a correct response, and the percentage of correct responses was calculated automatically with the use of ImageTM software.

### Y-maze test

Spatial working memory was evaluated on the basis of spontaneous alternation behavior in a Y-maze consisting of three arms (labeled A, B, and C) that diverged at an angle of 120 degrees from the central point (O’Hara & Co.). Each mouse was placed at the center of the maze and allowed to move freely for 10 min. An alternation was defined as consecutive entry into all three arms. For example, sequential entry into the arms in a pattern ABCBCBCA is counted as two alternations: the first consecutive ABC and the last consecutive BCA out of the six consecutive trios of arm choices. Maximum alternation was defined as the total number of arm entries minus 2. The percentage of alternations was calculated according to the following formula: 100 × (measured alternations/maximum alternations). The total number of arms entered during each session was also recorded. Data acquisition and analysis were performed automatically with ImageYM software.

### Electrophysiology

Electrophysiological experiments were performed as described previously [35–37]. WT and *PRT*^–/–^ mice at 8 to 11 weeks of age were subjected to analysis of LTP and the input–output relation, whereas those at 4 to 5 weeks of age were analyzed for LTD. Transverse hippocampal slices (thickness of 400 μm) were prepared with a tissue slicer (Vibratome 3000 from Lancer, St. Louis, MO, or LEICA VT 1200S from Leica Biosystems, Nussloch, Germany) in a Krebs–Ringer external solution that contained 119 mM NaCl, 2.5 mM KCl, 1 mM NaH_2_PO_4_, 26.2 mM NaHCO_3_, 11 mM glucose, 2.5 mM CaCl_2_, and 1.3 mM MgSO_4_ and was saturated with 95% O_2_ and 5% CO_2_. After recovery for at least 1 h at room temperature, a slice was transferred to the recording chamber. Synaptic responses were recorded in the stratum radiatum of the CA1 region with the extracellular field-potential recording technique and with the use of a glass recording pipette filled with 3 M NaCl. Experiments examining the input–output relation and LTP were performed at 25.0° ± 0.5 °C, whereas those examining LTD were performed at 34.0° ± 0.5 °C. The external solution for recordings was the same as that for slice preparation. For recordings of LTP and the input–output relation, picrotoxin (100 μM) (Sigma-Aldrich) was included in the external solution to inhibit γ-aminobutyric acid type A (GABA_A_) receptor–mediated inhibitory synaptic responses. For all recordings, the CA3 region was surgically cut off to prevent bursting activity. Schaffer collateral-commissural fibers were stimulated at 0.1 Hz (test pulse) with a bipolar tungsten electrode to evoke synaptic responses. With the exception of experiments examining the input–output relation, the stimulus strength was adjusted to evoke EPSPs with a slope value of 0.10 to 0.15 mV/ms. After a stable baseline was established, LTP was induced by stimulation at 100 Hz for 1 s, whereas paired-pulse stimulation (interpulse interval of 50 ms) at 1 Hz for 15 min was applied in the presence of 50 μM D-(–)-2-amino-5-phosphonopentanoic acid (D-AP5) (Tocris Bioscience) to induce LTD. Alternatively, 100 μM DHPG (Tocris Bioscience) was applied for 5 min to induce LTD. The input–output relation was examined in the presence of 50 μM D-AP5 to block *N*-methyl-D-aspartate (NMDA) receptor–mediated EPSPs. A low concentration (1 μM) of the non-NMDA receptor antagonist 6-cyano-7-nitroquinoxaline-2,3-dione (CNQX, Tocris Bioscience) was also added to the external solution in order to partially block AMPA receptor–mediated EPSPs and thereby allow more accurate measurements as a result of a reduction in the nonlinear summation of field EPSPs. EPSPs were evoked by stimulation of various strengths, and the data were first sorted by binning the fiber volley amplitudes, after which EPSP slopes were averaged within each bin. Synaptic responses were recorded with the use of MultiClamp 700B amplifiers (Molecular Devices, Sunnyvale, CA), and the signal was digitized at 10 kHz with Digidata 1440A (Molecular Devices), analyzed with pClamp10 (Molecular Devices), and stored on a personal computer.

### Data analysis

The applications for analysis of behavioral data (ImageBT, ImageLD, ImageEP, ImageSI, ImageHC, ImageCSI, ImagePS/TS, ImageFZ, ImageBM, ImageTM, ImageYM) were based on ImageJ (https://rsb.info.nih.gov/ij) and developed by T. Miyakawa [38]. Statistical analysis was performed with the use of SAS University Edition software (SAS Institute, Cary, NC). Normality of data was first assessed with the Shapiro–Wilk test, and homogeneity of variance between genotypes was examined with the F-test for each behavioral measure. If the normality assumption was not met, the Wilcoxon rank sum test was applied for comparisons between genotypes. If data were normally distributed and variance was homogeneous between genotypes, comparisons were performed with Student’s *t* test. If homogeneity of variance was not assumed, Welch’s *t* test was applied instead of Student’s *t* test. ANOVA was also conducted for all tests. All statistical analysis values, including ANOVA results, are included in Additional file 1: Table S1.

## Supplementary information

**Additional file 1: Table S1.** Statistical analysis of behavioral data in PRT knockout

## Data Availability

The datasets used and/or analyzed during the current study are available from the corresponding author on reasonable request.
